# Caffeine consumption and exposure in Saudi Arabia: a cross-sectional analysis

**DOI:** 10.3389/fnut.2025.1556001

**Published:** 2025-05-20

**Authors:** Ghadir Fallata, Omar Alhumaidan, Sarah Alkhunein, Nimah Baqadir, Rahaf Bin Sheehah, Faisal Binsunaid, Atheer Alragea, Mazen Alfayez, Nahla Bawazeer

**Affiliations:** ^1^Saudi Food and Drug Authority, Riyadh, Saudi Arabia; ^2^Public Health Authority, Riyadh, Saudi Arabia; ^3^Department of Health Sciences, College of Health and Rehabilitation Sciences, Princess Nourah Bint Abdulrahman University, Riyadh, Saudi Arabia

**Keywords:** caffeine, coffee, tea, Saudi coffee, soft drinks, caffeine consumption, caffeine exposure, caffeine intake levels in Saudi Arabia

## Abstract

**Introduction:**

Caffeine, a central nervous system stimulant, with 80% worldwide consumption. Despite its popularity, limited research has evaluated caffeine exposure in Saudi Arabia (SA). Given that excessive caffeine intake can cause symptoms such as increased heart rate, headaches, and hyperactivity, understanding community-level consumption patterns is critical. This study aimed to assess caffeine consumption and exposure among Saudi consumers by assessing caffeine consumption levels in the adult population of SA.

**Methods:**

A cross-sectional, Internet-based questionnaire study was conducted between September 2021 and January 2022. The questionnaire comprised 92 questions divided into three sections: demographic characteristics, a food frequency questionnaire on caffeine-containing products, and practices related to caffeine consumption.

**Results:**

A total of 1,039 participants were included in the study. The average daily caffeine exposure among Saudi adults was 218 mg/day. The highest caffeine exposure was from hot coffee (64.8 mL/day), soft drinks (41.9 mL/day), iced coffee (35.7 mL/day), tea (26.3 mL/day), energy drinks (16.5 mL/day), and chocolate (3.2 mg/day). This level of consumption falls within the recommended safe limit for healthy adults, which is 400 mg/day.

**Discussion:**

Caffeine consumption among Saudi individuals largely falls within the recommended safe range. Further research should investigate the long-term health effects of caffeine, emphasizing the need for public health initiatives to encourage recommended consumption.

## 1 Introduction

Caffeine is widely consumed worldwide. Caffeine consumption is estimated to be ~80% of total population (worldwide) (1). Caffeine is naturally present in various plant leaves and seeds, such as coffee beans, tea leaves, chocolate, and kola nuts. In addition, it is synthesized and added to medicines and certain food items such as carbonated drinks (2). The common name for caffeine is 1, 3, 7- trimethylxanthine (1), and it is one of the most widely used mood- and behavior-altering drugs (3).

Caffeine is considered as a central nervous system stimulant, and its consumption affects cognitive function in various ways. Caffeine provides most people a temporary energy boost and improves their mood (4). The caffeine short-term effects include better cognitive performance, improved alertness, and increased anxiety (4). Data have shown that an average adult's moderate daily caffeine intake of (400 mg/day) has not been linked to adverse effects on the human body (5). However, women of childbearing age and children may be at greater risk from caffeine (5). Moreover, according to scientific opinions on the safety of caffeine, a single dose of caffeine is up to (200 mg/day) in the general healthy adult population (6). In a recent biobank study in the United Kingdom, light-to-moderate caffeine consumption was associated with a lower risk of death (7).

In 2014, research indicates that ~85% of individuals in US regularly consume at least one type of caffeinated beverage daily, and the highest consumers (226 mg/day of caffeine) were between 50 and 64 years old. The primary contributor to caffeine consumption was coffee across all age groups, and the lowest consumption percentage was energy drinks in all age groups (8). (9) reported that 92% of students consumed caffeine, and coffee was the main caffeinated product in male consumers (120 mg/day) and female consumers (111 mg/day). Caffeine is consumed for various reasons, such as staying alert (79%), savoring the flavor (68%), socializing while drinking (39%), enhancing concentration (31%), and boosting physical energy (27%) (10).

In 2019, Agarwal et al. suggested caffeine consumption limit guidelines for overweight individuals. In Switzerland, Rochat et al. observed differences in mean caffeine consumption across different age groups; participants aged between 18 and 34 consumed 140 mg/day, and those aged between 50 and 64 consumed 228 mg/day (8). In New Zealand, four factors were identified in a qualitative study that impacted the intake of caffeinated products, including social and cultural context, environmental conditions, individual physical, psychological, and emotional expectations, and personal knowledge and perception (11). Watson et al. revealed that the study group that consumed 192.1 mg/day of caffeine reported significantly poorer sleep than those who reported good sleep quality (125.2 mg/day) (12).

A cross-sectional study conducted on students at North Lebanon University identified that the vast majority (97%) of students consumed caffeinated products. The main reasons for their caffeinated beverage consumption were to sense increased alertness, work long shifts, express a taste preference, treat headaches, enhance their mood, and diminish anxiety (13). At Zayed University in Dubai, the average intake of caffeinated beverages was 249.7 mg/day among students. In addition, 86% of participants consumed caffeinated beverages in both sexes. There was no statistical difference between males and females (35%) who consumed more than 400 mg/day. However, there was a statistically significant difference between caffeine and improved exercise performance in addition to caffeine and weight loss among males. They found that most caffeine intake comes from coffee, followed by tea, in both sexes (14). In the Kingdom of Bahrain, a study indicated that almost 98% of university students consumed caffeinated products, and caffeine consumption was 268 mg/day. In addition, it seemed males consumed more caffeine than females in Bahrain. Coffee, black tea, and energy drinks were the main caffeinated product consumed. Additionally, the results showed that the study group that consumed 400 mg/day or more was statistically significantly at high risk for symptoms, including headaches, episodes of terror or panic, sensations of being trapped or caught, excessive worrying, and feeling of worthlessness (15).

Limited studies have evaluated the caffeine intake in different regions of Saudi Arabia (SA). In Riyadh, a study reported ~43% consume < 300 mg/day of caffeine. The highest caffeine intake was detected in coffee consumers, followed by soft drink consumers, at ~94% and 91%, respectively, whereas the percentage of tea consumers was ~46%. Moreover, a significant correlation was found between a reduction in body weight and increased caffeine intake (16). Another study estimated the prevalence of energy drink among young adults and adolescents in Makkah, SA. Coffee consumers comprised ~88% of the participants, and coffee was consumed significantly more by overweight participants than by those of normal weight. In addition, ~47% were energy drink consumers; ~27% were drinking more than two cans per day, and a ~44% were drinking energy drinks during the examination period (17).

A study conducted in the Northern Border area of SA found that coffee was the most consumed caffeinated product (51%). Carbonated soft drinks ranked as the second most consumed product (23%). However, 39% of the participants had no reason for caffeine consumption; 31% consumed one cup/can daily, while 25% drank two cups/cans daily. Headache has been reported as the main symptom of quitting or delaying caffeine consumption. The cost of caffeine products was not a significant factor for 77% of the participants, 69% did not follow their friends' choice of caffeine type, and ~58% of the study group lacked information related to drug interactions with caffeine. Nevertheless, 77% of the study group believed overconsumption of caffeine badly influenced health (18). Moreover, another study stated that increasing daily caffeine consumption and maintaining a constant dosage of caffeine might benefit individuals in preventing or controlling fat accumulation and reducing the risk of diabetes (19).

## 2 Objective and scope

The objective of this study was to assess dietary caffeine consumption (intake) and exposure. This will be accomplished by gathering data on the frequency of dietary intake of potential caffeine sources. The results of this study will enable the assessment of whether caffeine consumption in Saudi Arabia conforms to the recommended health-based guideline value (HBGV).

## 3 Materials and methods

### 3.1 Study design

This cross-sectional, questionnaire-based Internet study was conducted from September 2021 to January 2022. This study was approved by the Ethics Research Committee of Princess Nourah Bint Abdulrahman University (Riyadh, SA) (IRB no. 21-0288, dated 13 June 2021). Informed consent was obtained from all participants. The confidentiality of the study participants' information was including names and other identifying information details ensured by storing it in password-protected files on secure servers. Access to the information was strictly limited, and no party was provided without the explicit written consent of the principal investigator of the study.

### 3.2 Sample size

Since there were no previous studies in SA that could be used as a reference to calculate the sample size needed to assess caffeine exposure among Saudis, we performed a sample size calculation based on the total adult population in Saudi Arabia (including both Saudis and non-Saudis) which was reported to be 25,828,206 (aged 15 years or older) by the Saudi General Authority for Statistics in 2018 by using an online Epi Info sample size calculator. For calculation parameter, a confidence level of 99%, and margin of error of 5%. For responses rate, we assumed that 50% of the target subjects would agree to participate in the study. Therefore, the total calculated sample size was 1,083 participants.

### 3.3 Participants

A convenience sample of 1,083 adult residents and citizens aged 15 and above in Saudi Arabia of Saudi Arabia was included in the study. The participants were requested to complete a publicly distributed electronic questionnaire created using a Microsoft form that required responses to all questions before submission, which ensured there was no missing data. The electronic questionnaire was dispersed using snowball sampling on social media platforms including Twitter and WhatsApp. The National Nutrition Committee's Twitter account shared the survey link, and the link of questionnaire also sent to personal contacts in the authors' WhatsApp accounts, including family members, friends, and neighbors. These individuals were encouraged to forward the link to their contacts within Saudi Arabia, and the link was shared among numerous WhatsApp social groups.

The inclusion criteria for this study were adults aged 15 years and above, and residents and citizens of Saudi Arabia. The exclusion criteria were non-Saudi residents or non-citizens of Saudi Arabia, individuals under the age of 15, and those who did not complete the questionnaire.

### 3.4 Study instruments

The questionnaire included 92 questions that take ~10–15 min to answer, as determined through pilot testing where participants completed it within this timeframe on average. The questionnaire contained several sections: (1) demographic characteristics to measure general characteristics [age, sex, marital status, female care status, work status, education level, monthly income, and subjective height (cm), weight (kg), and calculated body mass index (kg/m2)]; (2) a food frequency questionnaire on caffeine products (FFQ-C) to collect caffeine consumption data. The FFQ-C was adopted from the 2010 Caffeine Consumption Questionnaire of the University of North Carolina Wilmington (20) and modified according to Saudi consumption (including caffeine products with food item lists that are prevalent); (3) questions related to knowledge, beliefs, and practices (such as time of consumption and adding sugar and additive flavors to tea and coffee) (see Supplementary material 1 for an overview of FFQ-C structure).

**Figure 1:**
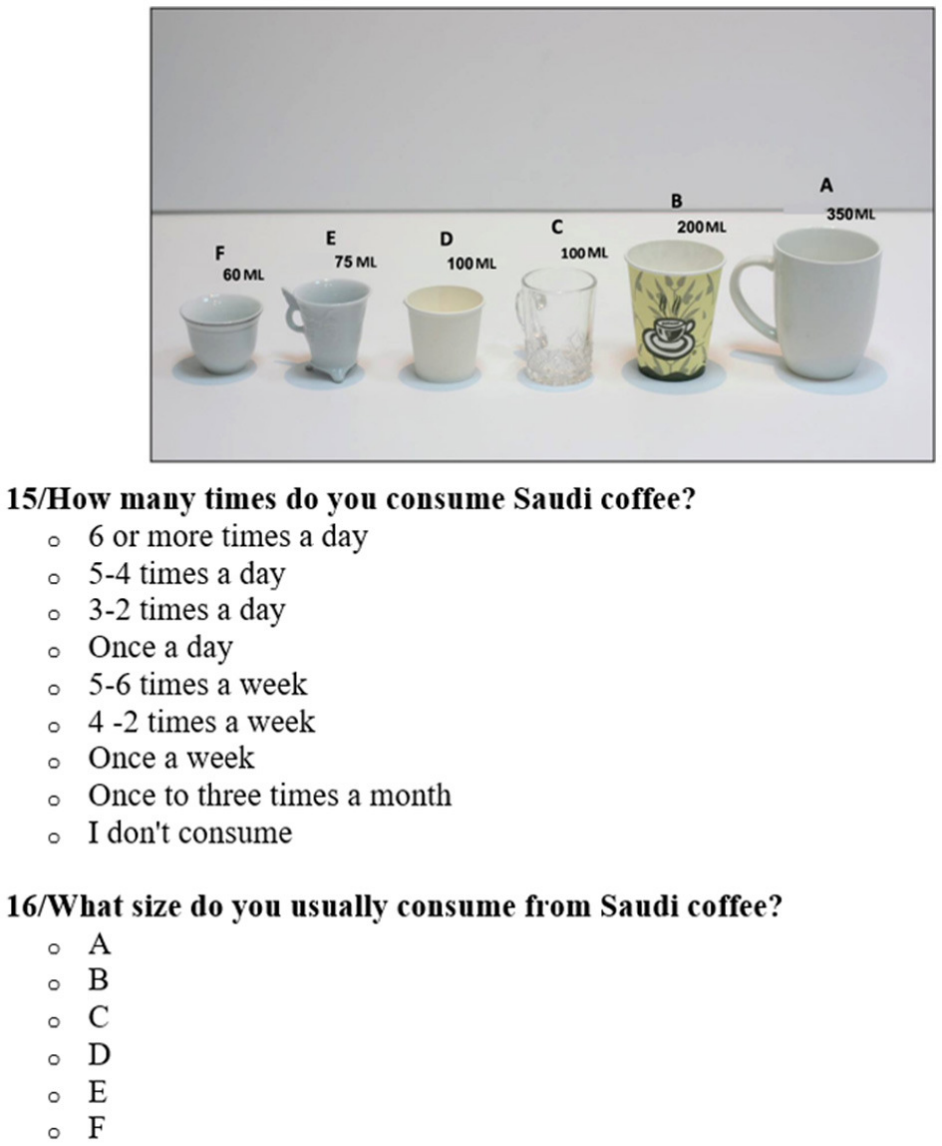
Example of caffeine product (Saudi coffee) frequency and portion sizes question from the food frequency questionnaire on caffeine products (FFQ-C).

For a better illustration of portion sizes, the FFQ-C had a list of 29 caffeinated foods and drinks: [coffee (15 items), tea (six items), soft drinks (two items), energy drinks (one item), and chocolate (five items)] supported with photos of different volume consumption for each product to choose from. For each food item (e.g., Saudi coffee, black coffee, tea, and chocolate), participants were asked to report the frequency of consumption using the following options: six times or more per day, 4–5 times per day, 2–3 times per day, once per day, 5–6 times per week, 2– response rate 4 times per week, once per week, 1–3 times per month, or not consumed at all. They were also asked to indicate portion size, categorized from A (350 ml) to F (60 ml) (Figure 1). The portion sizes were determined using the most commonly used utensils among the Saudi population, based on the type of drinks consumed [Figure 1; see Supplementary material 2 for an overview of additional results (portion sizes and frequency options of caffeine products)].

For validity of the instrument, the questionnaire was shared and reviewed by five experts in Nutrition and Dietetics. Followed by carried out a pilot study with 50 participants to empirically test the questionnaire, refine the food list, and enhance the clarity of the questions. In future surveys, the classification criteria for variables such as income, education level, and employment status will be refining to enhance the accuracy of comparisons.

Since most food composition databases have limited brand specificity, caffeine values were obtained from multiple sources, including companies and brands' websites, scientific literature (21), and laboratory analysis (described later in the laboratory analysis section) at the Saudi Food and Drug Authority Laboratory (SFDA-Lab); practice questions were developed by researchers.

### 3.5 Caffeine content

The caffeine content of the FFQ-C list of 29 caffeinated foods and drinks was determined by different sources, including companies and brand websites or SFDA-Lab and calculated per 100 mL (see Supplementary material 2). Since most food and drink products (except Saudi and Turkish coffee) were prepared by companies and brands themselves, the value of caffeine content was reliably obtained from the companies, brands, and websites.

For espresso products, the value of caffeine is very broad for different companies and brands. Therefore, caffeine values were obtained from a study that included 20 different brands, and the collection and analysis methods were clear (22).

In terms of Saudi and Turkish coffee caffeine values, and because they need to be prepared (cooked) and then analyzed, the reference was obtained from SFDA-Lab (23) [see Supplementary material 2 for an overview of additional results (caffeine content and references)].

### 3.6 Caffeine consumption

Caffeine consumption (intake) of products was determined by multiplying the average consumption of caffeine by the caffeine content of product [see Supplementary material 2 for an overview of additional results (caffeine content and references)]. Additionally, caffeine intake for product categories was calculated as the average caffeine content across all products within the category.

The mean consumption was assessed using the FFQ-C, expressed in milliliters or milligrams, and then multiplied by the caffeine content to provide an accurate estimate of dietary caffeine consumption.

### 3.7 Caffeine exposure

Caffeine exposure was calculated by measuring the amount of caffeine consumed by individuals in their diet that was intentionally added or unintentionally presents (21). Equation 1 measured the volume consumed from the FFQ-C in milliliters or milligram multiplied by the caffeine content in order to state the accurately evaluate dietary exposure to caffeine.


(1) $$\begin{array}{l} \text{Dietary exposure }=\sum \text{Concentration of chemical in food}\\ \;\times \text{ Food consumption}/\text{Body weight }\left(\text{kg}\right) \end{array}$$

This process aligns with the guidelines provided by the Food and Agriculture Organization (22). In standard assessments, the resulting products of concentration and consumption are typically divided by body weight. However, in the case of caffeine, whose HBGV is expressed in milligrams per day rather than milligrams per kilogram per day, the calculation is simplified to merely multiplying the caffeine concentration by the amount consumed (24). This approach directly compares the measured caffeine exposure and the established HBGV.

### 3.8 Statistical analysis

The data were verified and entered into a computer system using standardized codes written in an SPSS file. Data analysis was conducted using SPSS software (version 23). Data are presented as frequency and proportion. The collected data were analyzed to determine the patterns of caffeinated food consumption in the SA population. Descriptive statistics (mean, frequency, and proportion) were calculated to summarize the data. To measure the amount of caffeine exposure, total caffeine intake was calculated based on the reported frequency and amount of consumption of caffeinated foods. The caffeine content of each food item was obtained from foo composition tables developed in this study [see Supplementary material 2 for an overview of additional results (caffeine content and references)].

### 3.9 Data uncertainty

Following the determination of the mean, minimum, and maximum values for each beverage based on the survey results, the total exposure was calculated. When calculating the total exposure, it is necessary to consider that beverages may not be consumed on the same day, and consumption patterns exhibit variability. Different beverage choices are likely to be consumed in various manners. To address this variability, a Probabilistic Approach was employed based on the exposure report from the WHO Exposure Guidelines (21). This approach initially obtains a random sample from the survey population, and subsequently generates numerous simulated samples by conducting repeated sampling with replacement from the original sample. From these simulated samples, a confidence interval for the sample statistic of interest was constructed using the sampling distribution formed by the simulated samples. In this study, total exposure was based on the food item groups (Hot coffee, Iced coffee, Tea, Soft drinks, Energy drinks, and Chocolate) identified in the survey. The maximum, minimum, and mean values are calculated. For each beverage, this was represented as a triangular probability distribution. Six triangular distributions were utilized in a simulation of 10,000 iterations, where each iteration represented one day, resulting in a total of ~60, 000 iterations. The daily averages for caffeine consumption and caffeine intake were calculated for each day. Subsequently, the results demonstrated all the potential combinations of caffeine consumption.

## 4 Results

### 4.1 Participant characteristics

Of 1,039 participants, 996 (95.9%) Saudi and 43 (4.1%) non-Saudi were enrolled from different SA regions (61.5% from central, 20.8% from western, 9.9% from eastern, 4.1% from southern, and 3.7% from northern regions). Moreover, following inclusion criteria, both adult sexes aged ≥15 years old, 314 (30.2%) were male, and 725 (69.8%) were female. The male-to-female ratio was ~1:2.3. Overall, 40% of participants had a normal body mass index (BMI); 31% were overweight, 16.7% were obese I, 5% underweight, 4.2% were obese II, and 2.9% were obese III. Additional descriptive sociodemographic details such as educational background, marital and employment status, and monthly income are presented in Table 1.

**Table 1:** Demographic and socioeconomic characteristics of participants (*N* = 1,039).

**Variable**	**Category**	**Number of participants (*N*)**	**Percentage (%)**
Nationally	Saudi	996	95.9
	Non-Saudi	43	4.1
Region	Central region	639	61.5
	Eastern region	103	9.9
	Western region	216	20.8
	Northern region	38	3.7
	Southern region	43	4.1
Age range (Years)	15–19	37	3.6
	20–29	380	36.5
	30–39	265	25.5
	40–49	185	17.8
	50–59	130	12.5
	≥60	42	4.1
Gender	Male	314	30.2
	Female	725	69.8
Marital status	Single	446	42.9
	Married	547	52.6
	Divorced	33	3.2
	Widowed	13	1.3
Female care status	Pregnant	12	1.2
	Breastfeeding	8	0.8
Educational level	Less than high	33	3.2
	High school	148	14.2
	Bachelor's degree	640	61.6
	High than bachelors	218	21
Work status	Student	196	18.9
	Searching for job	106	10.2
	Employee	514	49.5
	Retired	79	7.6
	Self-employment	33	3.20
	Housewife	111	10.7
Income monthly (SR)	< 2,000	276	26.9
	2,000–5,000	160	15.4
	5,000–7,000	78	7.5
	7,000–10,000	127	12.2
	>10,000	398	38.3
BMI	Underweight	52	5
	Normal	416	40
	Overweight	322	31
	Obese type 1	174	16.7
	Obese type 2	44	4.2
	Obese type 3	31	2.9

### 4.2 Caffeine consumption

The top five highest mean consumption of caffeinated products among the participants was from Sugar-sweetened soft drinks (179.7 mL/day), black coffee (148 mL/day), hot tea (129.6 mL/day), Saudi coffee (106.7 mL/day), and iced black coffee (106.6 mL/day). The lowest mean consumption of chocolate powder (1.2 mL/day) was observed (Table 2).

**Table 2:** Mean caffeine consumption (mL or mg/day) for Saudi individuals from detailed caffeinated products that include food and drinks items.

**Food item**	**Mean consumption**		
	**Means**	**Min**	**Max**
**Hot coffee**	84.5 mL/day	0	410 mL/day
Saudi coffee	106.7 mL/day	0	350 mL/day
Turkish coffee	35.9 mL/day	0	350 mL/day
Black coffee	148 mL/day	0	500 mL/day
Espresso	49.3 mL/day	0	350 mL/day
Coffee–Flavored with milk	82.8 mL/day	0	500 mL/day
**Iced coffee**	57.2 mL/day	0	394.4 mL/day
Iced black coffee	106.6 mL/day	0	500 mL/day
Ice espresso or Espresso con panna	17.6 mL/day	0	350 mL/day
Iced coffee–Flavored with milk	47.5 mL/day	0	333.3 mL/day
**Tea**	94.3 mL/day	0	425 mL/day
Hot tea	129.6 mL/day	0	350 mL/day
Iced tea	59.1 mL/day	0	500 mL/day
**Soft drinks**	141 mL/day	0	900 mL/day
Sugar-sweetened beverages	179.7 mL/day	0	900 mL/day
No sugar beverages	102.3 mL/day	0	900 mL/day
**Energy drinks**	52.4 mL/day	0	330 mL/day
Energy drink	52.4 mL/day	0	330 mL/day
**Chocolate**	15 mg/day	0	129 mg/day
Dark chocolate	13.7 mg/day	0	57.5 mg/day
Chocolate with milk	20.1 mg/day	0	57.5 mg/day
Spreadable chocolate	2.5 mg/day	0	15 mg/day
Chocolate powder	1.2 mg/day	0	15 mg/day
Hot chocolate	37.5 mg/day	0	500 mg/day

The data distribution is asymmetrical, therefore median, maximum, and minimum values were incorporated in order to define consumption.

Regarding caffeine categories, the participants had the highest mean consumption from Soft drinks (141 mL/day), followed by tea (94.3 mL/day), hot coffee (84.5 mL/day), iced coffee (57.2 mL/day), energy drinks (52.4 mL/day), and lastly chocolate (15 mg/day).

### 4.3 Caffeine exposure

According to the result, the participants had high exposure to caffeine from hot coffee (64.8 mL/day), soft drinks (41.9 mL/day), iced coffee (35.7 mL/day), tea (26.3 mL/day), energy drinks (16.5 mL/day), and chocolate (3.2 mg/day) (Table 3).

**Table 3:** Mean total caffeine exposure (mL or mg/day) from detailed caffeinated products that include food and drinks items.

**Food item**	**Caffeine exposure**		

	**Means**	**Min**	**Max**
**Hot coffee**	64.8 mL/day	0	204.3 mL/day
Saudi coffee	53.2 mL/day	0	174.4 mL/day
Turkish coffee	46.6 mL/day	0	174.4 mL/day
Black coffee	116.7 mL/day	0	249.2 mL/day
Espresso	70.2 mL/day	0	174.4 mL/day
Coffee–Flavored with milk	37.1 mL/day	0	249.2 mL/day
**Iced coffee**	35.7 mL/day	0	308.1 mL/day
Iced black coffee	83.2 mL/day	0	390.5 mL/day
Ice espresso or Espresso con panna	6.3 mL/day	0	273.4 mL/day
Iced coffee–Flavored with milk	17.4 mL/day	0	260.3 mL/day
**Tea**	26.3 mL/day	0	134.2 mL/day
Hot tea	40.9 mL/day	0	110.5 mL/day
Iced tea	11.6 mL/day	0	157.9 mL/day
**Soft drinks**	41.9 mL/day	0	353.3 mL/day
Sugar-sweetened beverages	70.5 mL/day	0	353.3 mL/day
No sugar beverages	13.2 mL/day	0	353.3 mL/day
**Energy drinks**	16.5 mL/day	0	104 mL/day
Energy drink	16.5 mL/day	0	104 mL/day
**Chocolate**	3.2 mg/day	0	72.2 mg/day
Dark chocolate	7.6 mg/day	0	32.2 mg/day
Chocolate with milk	4 mg/day	0	32.2 mg/day
Spreadable chocolate	0.2 mg/day	0	8.4 mg/day
Chocolate powder	2.7 mg/day	0	8.4 mg/day
Hot chocolate	1.4 mg/day	0	280 mg/day

The data distribution is asymmetrical, therefore median, maximum, and minimum values were incorporated in order to define exposure.

**Figure 2:**
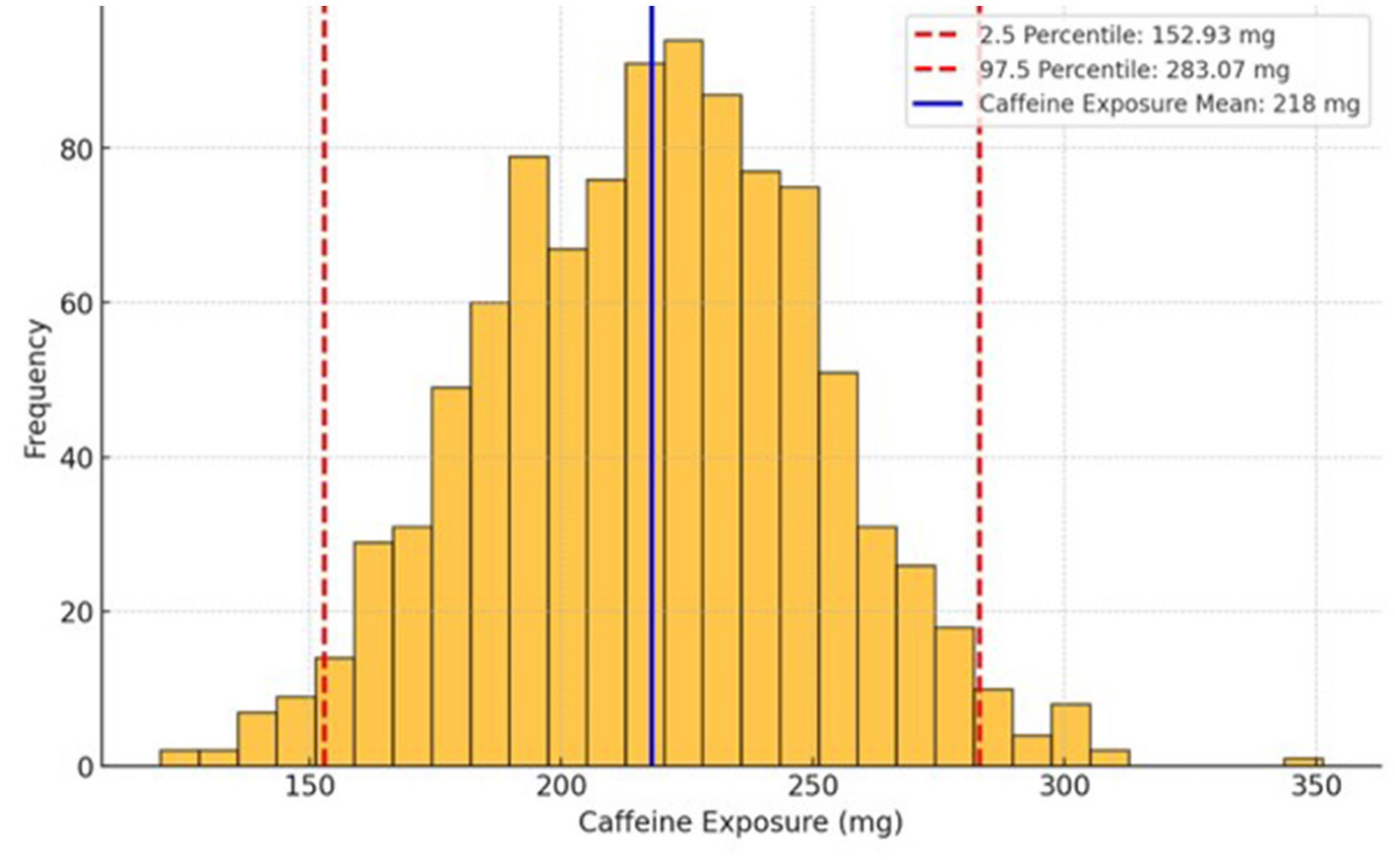
Average caffeine intake of Saudi participants. The intake has been calculated from the caffeine frequency and exposure of study participants.

Following the bootstrapping process and subsequent analysis of mean caffeine exposure of the participants to caffeine products (food and drinks) and potential combinations across days. The results indicated that the anticipated caffeine intake averaged 218 mg, with a range spanning from 152.93 mg to 283.07 mg (Figure 2).

There was an assessment for additive flavors (milk, sugar, artificial sweeteners, and honey), participants perception of whether it was important to display the caffeine content of food and beverages on food labels and restaurant menus, and time of caffeine consumption in the FFQ-C questionnaire [see Supplementary material 2 for an overview of additional results (frequency and percentage (%) of additive flavor, participants perception on caffeine legislation, and participants' daily period of caffeine consumption)].

## 5 Discussion

The US Food and Drug Administration recommended 400 mg of caffeine per day for all healthy adults (25). Few studies have evaluated caffeine intake in different regions of Saudi Arabia. In the Jazan region, Alfaifi et al. found that 20% of the participants exceeded the recommended level of caffeine (>400 mg/day) (26). In contrast, according to the results of this study, total caffeine consumption was 218 mg/day. Similarly, US consumption in patients aged between 50–64 years old (226 mg/day) (25). In Switzerland, Rochat et al. (8) reported differences in caffeine consumption of the Swiss population across age groups; participants aged 18–34 consumed ~140 mg/day, while those aged 50–64 consumed 228 mg/day. In New Zealand, a study showed that most participants (99.1%) consume ~146.73 mg/day of caffeine (3). Regionally, in the United Arab Emirates, the total caffeine consumed in the population is 316.7 mg/day (27). A study in Kingdom of Bahrain indicated that almost 98% of university students consumed caffeinated products, and caffeine consumption was 268 mg/day (15).

Several studies have reported a high consumption of sugar-sweetened beverages (SSBs) among the Saudi population. These findings revealed that demographic factors such as age, gender, educational background, and marital status were positively linked to SSB consumption (28). Our findings support previous studies in SA, which reported that adults aged 25 to 34 years have high SSB consumption (29). Moreover, Saudis consume more coffee than other drinks, reflecting the historical value of coffee in this population. Coffee is served on most Saudi occasions and at gatherings (30). In the present study, the consumption of black and iced coffee was higher than others, which could be explained by the data published by the World Coffee Portal, which shows that the coffee shop market grew 18.5% over the last 12 months (31). Regarding the low consumption of energy drinks, the SFDA issued a regulation that forces beverage manufacturers to declare warning statements on the label and place visible warning signs on shelves similar to those used on the label.

This study has shown that almost 98% of participants added white sugar to their beverages. Excess consumption of free sugars has been linked to various diseases and adverse health conditions, which are regrettably increasing in prevalence in many countries (32). It is contributing to weight gain and increases the risk of type 2 diabetes, chronic and heart diseases, and dental caries (32). A risk communication approach for educating consumers about the adverse health effects of sugar should be considered. However, low-fat milk is preferable to whole milk. Low-fat and skim milk have fewer calories and almost the same amount of vitamins as whole milk (national regulations mandate the fortification of milk with vitamins). Additionally, they contained lower amounts of saturated fat. According to the American Heart Association, saturated fatty acids have been shown in several studies to increase the risk of heart disease (33).

Based on the available evidence, caffeine short term effect can cause issues with the central nervous system, such as disrupted sleep, anxiety, and changes in behavior. Additionally, excessive caffeine consumption over a long period may lead to cardiovascular problems and stunted fetal development in pregnant women (34). Exceeding the caffeine intake limit may have adverse health effects. Almost 47% of the participants drank coffee before bedtime. In 2023, a study concluded that evening caffeine consumption shortened total sleep duration by 45 min, reduces sleep efficiency by 7%, delayed sleep onset by 9 min, and extended wakefulness after sleep onset by 12 min (35).

The strength of this study lies in addressing data uncertainty. On the other hand, several limitations were acknowledged that include, self-reporting that may threaten the reliability and validity of the data. Even though, self-reported data are cost-effective and direct methods for collecting data from large populations across large countries.

## 6 Conclusions

Understanding dietary caffeine consumption is essential for evaluating potential health risks and ensuring adherence to recommended intake levels. This study aimed to assess dietary caffeine consumption and exposure, including coffee, soft drinks, tea, etc. among Saudi individuals by analyzing the frequency of intake from various dietary sources. The investigation of the total consumption of Saudi individuals and the findings of this study provide insights into caffeine consumption, which was within the normal range and did not exceed the recommended level for healthy adults (400 mg/day).

However, given individual variations in caffeine sensitivity and consumption patterns, further research is necessary to explore potential long-term health effects and establish a more comprehensive risk-benefit analysis. Future studies should aim to expand the sample size and consider additional demographic and lifestyle factors that may influence caffeine consumption and health impact. Additionally, the study provides valuable insights for future public health initiatives, including targeted educational programs that promote adherence to dietary caffeine recommendations.

## Data Availability

The original contributions presented in the study are included in the article/Supplementary material, further inquiries can be directed to the corresponding author.
