# Supplementary material for: Caffeine consumption and exposure in Saudi Arabia: a cross-sectional analysis
Source: Front Nutr. 2025 May 20;12:1556001. doi: 10.3389/fnut.2025.1556001 (PMC12129793; doi:10.3389/fnut.2025.1556001)
Supplement: Supplementary file 1 [file Data_Sheet_1.pdf]

An Overview of Food Frequency Questionnaire on Caffeine Products (FFQ-C) Structure

(After translation form Arabic to English)

Survey about the amount of dietary caffeine consumption by individuals in the Kingdom of Saudi Arabia

You are in the process of participating in a survey that complements a study entitled (Knowledge of the food consumption of individual caffeine in the Kingdom of Saudi Arabia) presented by the National Committee on Nutrition in cooperation with the Saudi Food and Drug Authority and the Faculty of Health and Rehabilitation Sciences at Princess Noura bint Abdulrahman University.

Scope of study: Foods and drinks containing caffeine.
Objective of the study: Assessing caffeine consumption levels among the adult population in the Kingdom.
Target group: Participants aged 15 years and above.
Time taken to answer the survey: 15-20 minutes.

Information revealing your identity will not be requested directly and all data will be treated in complete privacy

Approval: Please select one of the options below:

- I have read the information above and agree to participate by filling out the questionnaire
- I do not agree to fill out the questionnaire (when choosing this option, you will leave the survey)

1/Are you a resident of Saudi Arabia?

- Yes
- No

2/Gender

- Male
- Female

3/Are you pregnant?

- Yes
- No

4/Are you breastfeeding?

- Yes
- No

5/Age

………..

6/Weight (kg)

………..

7/Height (cm)

……….

8/Nationality

- Saudi
- Non-Saudi

9/Marital status

- Single
- Married
- Divorced
- widow

10/Educational level

- Uneducated
- Diploma
- Bachelor’s degree

- Postgraduate (after bachelor's degree)

11/Job status

- Student
- Employed
- Unemployed (freelance)
- Unemployed (looking for a job)
- Retired
- Unemployed (housewife)

12/Your monthly income

- Less than 2000 Saudi riyals
- Between 2000 to 5000 Saudi riyals
- More than 5000 to 7000 Saudi riyals
- More than 7000 to 10000 Saudi riyals
- More than 10000 Saudi riyals

13/Region

- Central Region
- Northern Region
- Southern Region
- Western Region
- Eastern Region

14/Have you been diagnosed by your doctor with one of the following conditions?

(You can choose more than one answer)

- No, I have not been diagnosed
- Cardiovascular diseases (e.g. high blood pressure, atherosclerosis, etc.)
- Cancer
- Diabetes
- Kidney disease
- Liver diseases
- Stomach ulcer
- Anemia (due to low iron)
- Bone fragility (softness) (due to vitamin D deficiency)
- Other ….

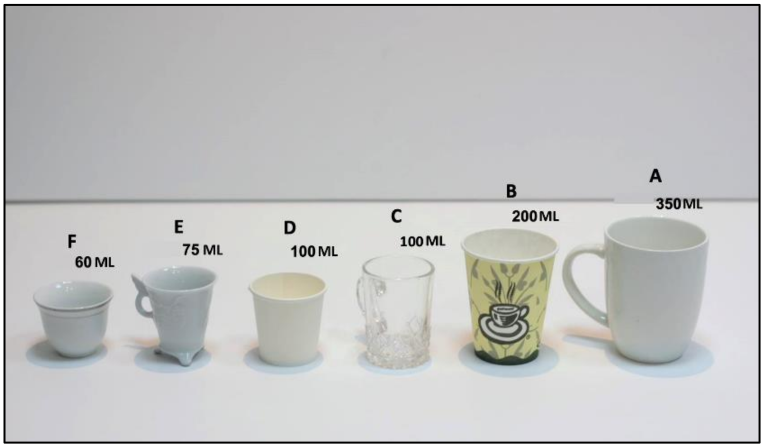

15/How many times do you consume Saudi coffee?

- 6 or more times a day
- 5-4 times a day
- 3-2 times a day
- Once a day
- 5-6 times a week
- 4 -2 times a week
- Once a week
- Once to three times a month
- I don't consume

16/What size do you usually consume from Saudi coffee?

- A
- B
- C
- D
- E
- F

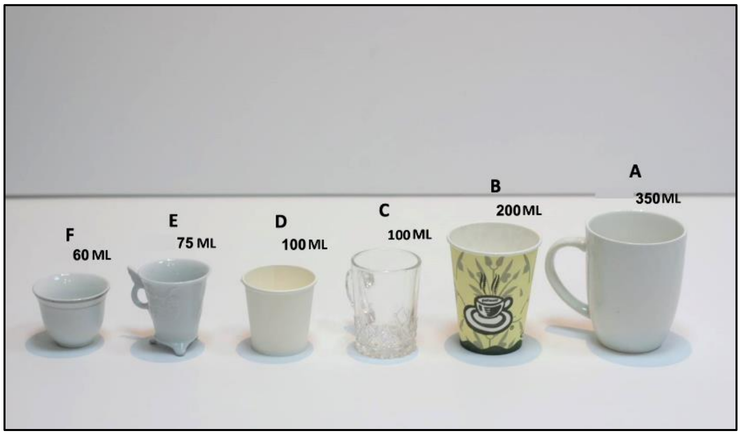

17/How often do you consume Arabic instant coffee?

- 6 or more times a day
- 5-4 times a day
- 3-2 times a day
- Once a day
- 5-6 times a week
- 4 -2 times a week
- Once a week
- Once to three times a month
- I don't consume

18/ What size do you usually consume from Arabic instant coffee?

- A
- B
- C
- D
- E
- F

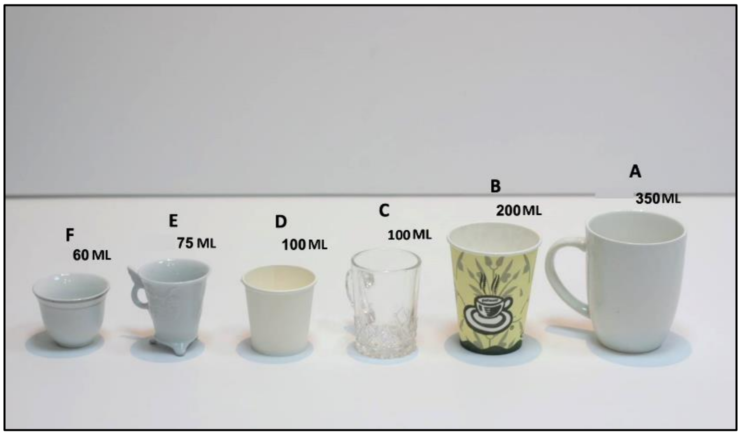

19/How often do you consume Turkish coffee?

- 6 or more times a day
- 5-4 times a day
- 3-2 times a day
- Once a day
- 5-6 times a week
- 4 -2 times a week
- Once a week
- Once to three times a month

- I don't consume

20/What size do you usually consume from Turkish coffee?

- A
- B
- C
- D
- E
- F

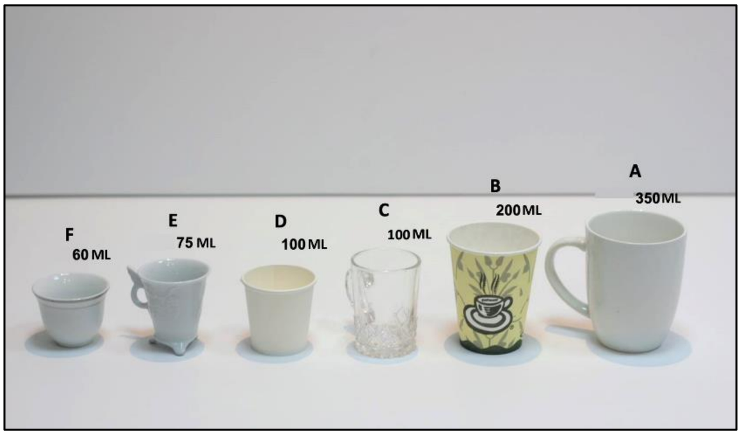

21/How often do you consume espresso?

- 6 or more times a day
- 5-4 times a day
- 3-2 times a day
- Once a day
- 5-6 times a week
- 4 -2 times a week
- Once a week
- Once to three times a month
- I don't consume

22/What size do you usually consume from espresso?

- A
- B
- C
- D
- E
- F

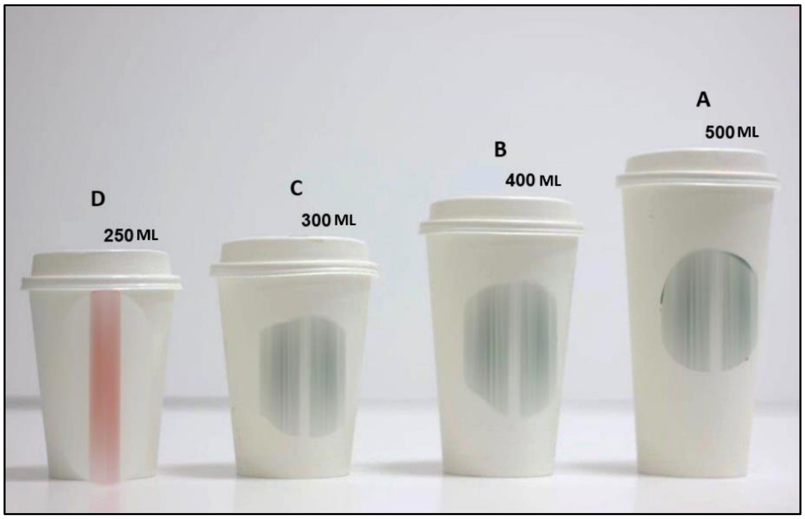

23/How often do you consume decaffeinated coffee?

- 6 or more times a day
- 5-4 times a day
- 3-2 times a day
- Once a day
- 5-6 times a week
- 4 -2 times a week
- Once a week
- Once to three times a month
- I don't consume

24/What size do you usually consume from decaffeinated coffee?

- A
- B
- C
- D

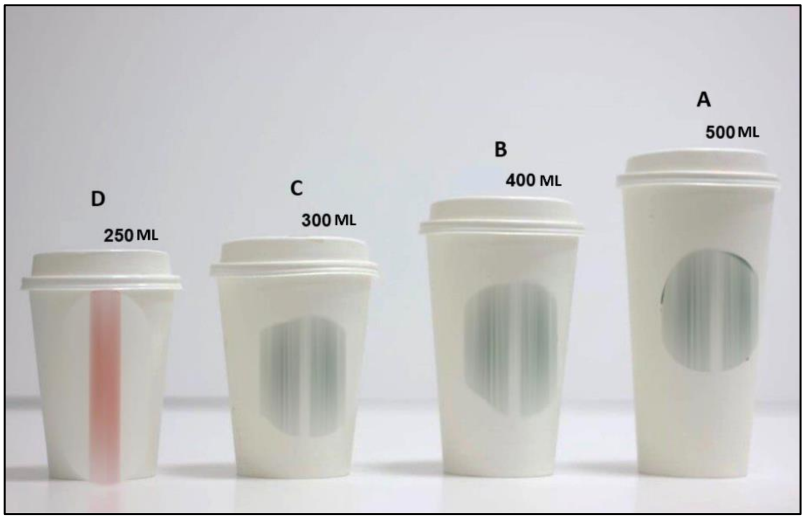

25/How often do you consume hot black coffee (Americano)?

- 6 or more times a day
- 5-4 times a day
- 3-2 times a day
- Once a day
- 5-6 times a week
- 4 -2 times a week
- Once a week
- Once to three times a month
- I don't consume

26/What size do you usually consume from hot black coffee (Americano)?

- A
- B
- C
- D

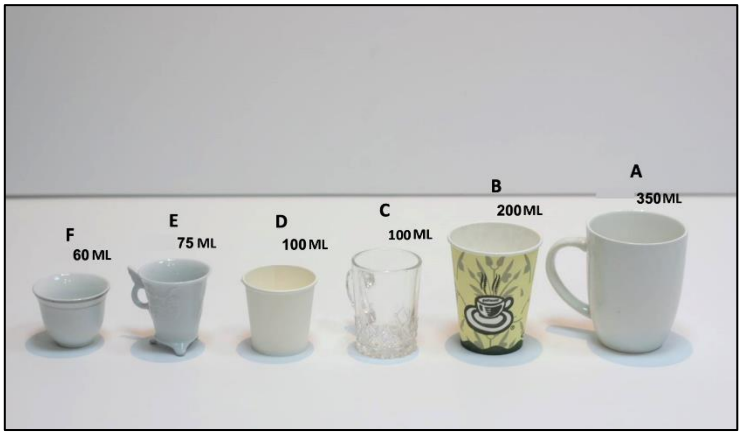

27/How often do you consume hot instant black coffee?

- 6 or more times a day
- 5-4 times a day
- 3-2 times a day
- Once a day
- 5-6 times a week
- 4 -2 times a week
- Once a week
- Once to three times a month
- I don't consume

28/What size do you usually consume from hot instant black coffee?

- A
- B
- C
- D
- E
- F

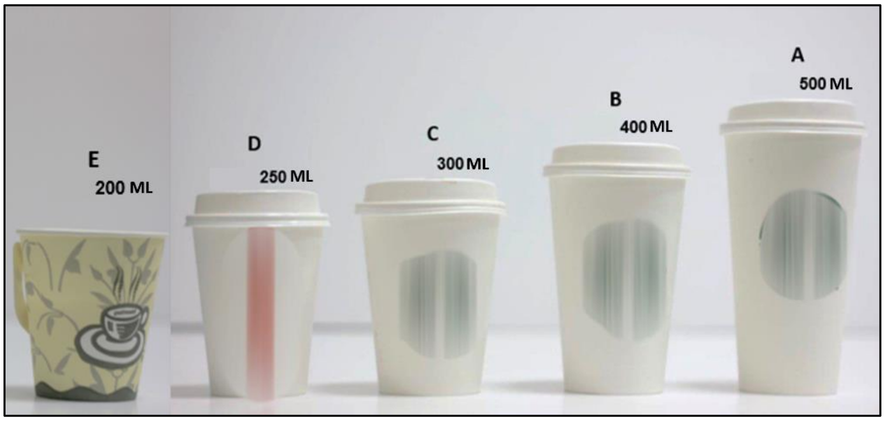

29/How often do you consume hot cappuccino?

- 6 or more times a day
- 5-4 times a day
- 3-2 times a day
- Once a day
- 5-6 times a week
- 4 -2 times a week
- Once a week
- Once to three times a month
- I don't consume

30/What size do you usually consume from hot cappuccino?

- A
- B
- C
- D
- E

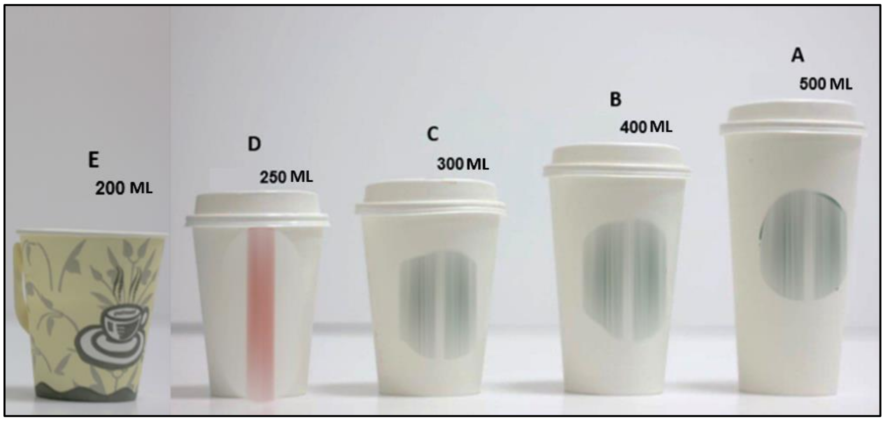

31/How often do you consume hot latte?

- 6 or more times a day
- 5-4 times a day
- 3-2 times a day
- Once a day
- 5-6 times a week
- 4 -2 times a week
- Once a week
- Once to three times a month
- I don't consume

32/What size do you usually consume from hot latte?

- A
- B
- C
- D

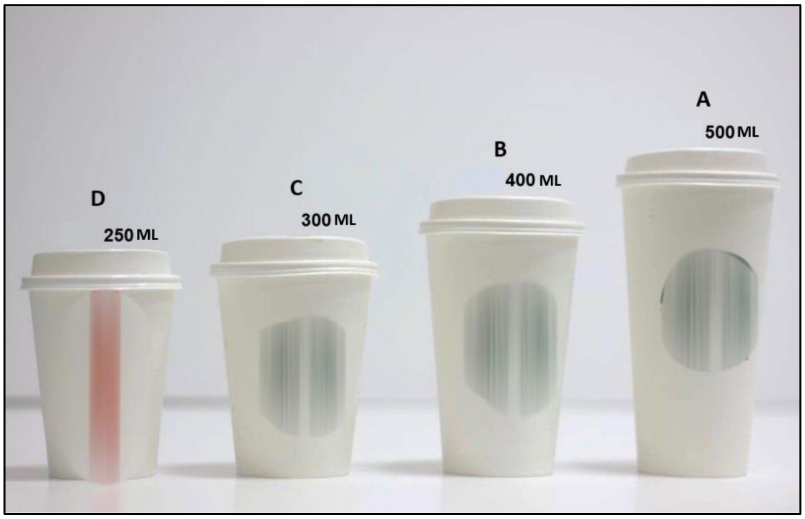

33/How often do you consume caramel maketo or hot spinach latte?

- 6 or more times a day
- 5-4 times a day
- 3-2 times a day
- Once a day
- 5-6 times a week
- 4 -2 times a week
- Once a week
- Once to three times a month
- I don't consume

34/What size do you usually consume from caramel maketo or hot spinach latte?

- A
- B
- C
- D

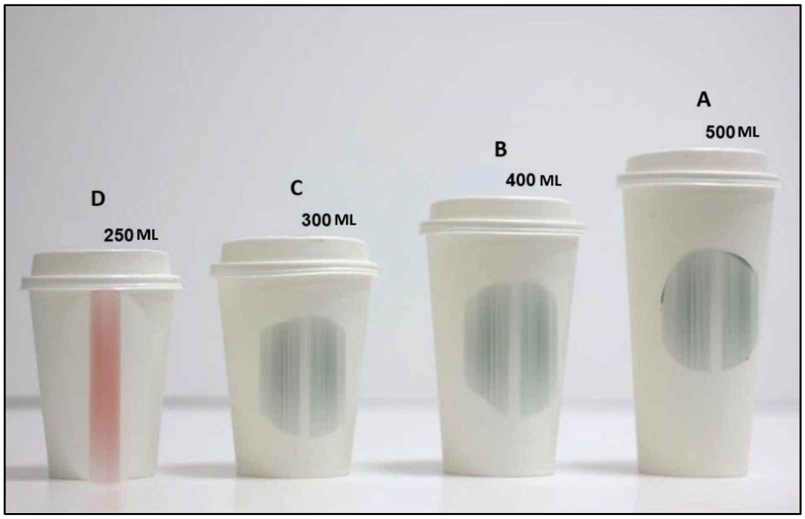

35/How often do you consume mocha or hot white chocolate mocha?

- 6 or more times a day
- 5-4 times a day
- 3-2 times a day
- Once a day
- 5-6 times a week
- 4 -2 times a week
- Once a week
- Once to three times a month
- I don't consume

36/What size do you usually consume from mocha or hot white chocolate mocha?

- A
- B
- C
- D

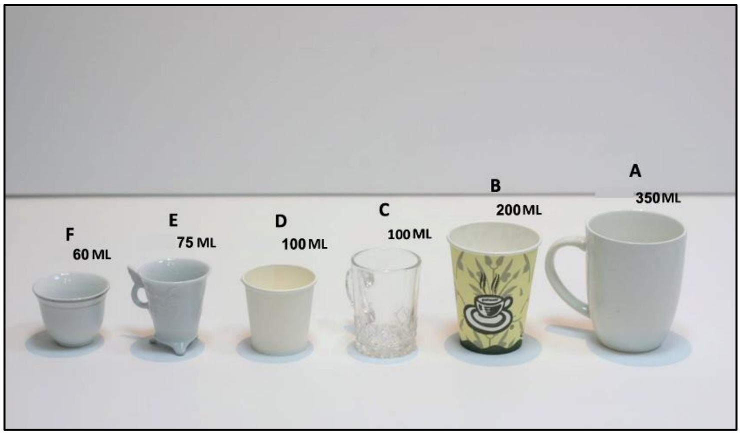

37/How often do you consume espresso or iced con panna espresso?

- 6 or more times a day
- 5-4 times a day
- 3-2 times a day
- Once a day
- 5-6 times a week
- 4 -2 times a week
- Once a week
- Once to three times a month
- I don't consume

38/What size do you usually consume from espresso or iced con panna espresso?

- A
- B
- C
- D
- E
- F

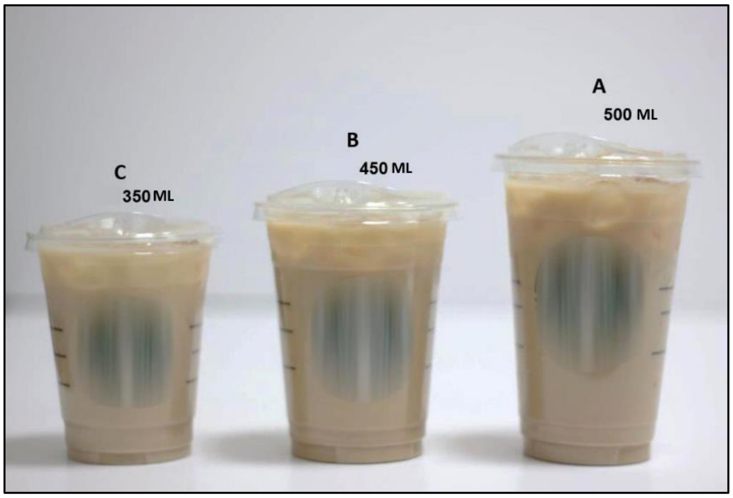

39/How often do you consume iced coffee, iced Americano, or Cold Brew?

- 6 or more times a day
- 5-4 times a day
- 3-2 times a day
- Once a day
- 5-6 times a week
- 4 -2 times a week
- Once a week
- Once to three times a month
- I don't consume

40/What size do you usually consume from iced coffee, iced Americano or Cold Brew?

- A
- B
- C

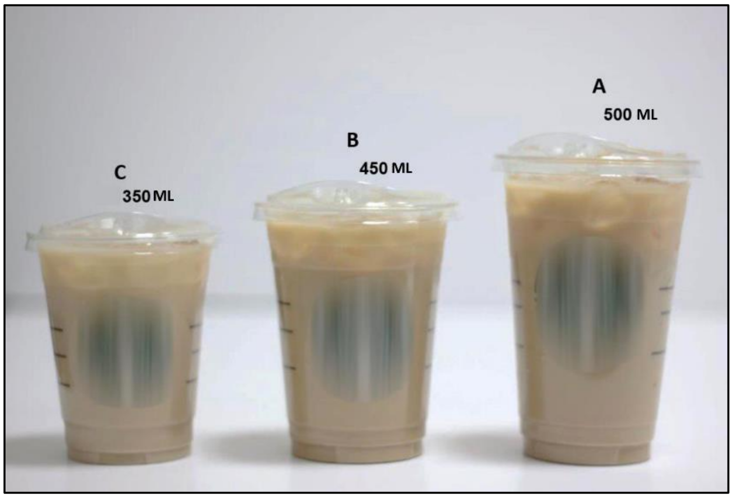

41/How often do you consume iced latte?

- 6 or more times a day
- 5-4 times a day
- 3-2 times a day
- Once a day
- 5-6 times a week
- 4 -2 times a week
- Once a week
- Once to three times a month
- I don't consume

42/What size do you usually consume from iced latte?

- A
- B
- C

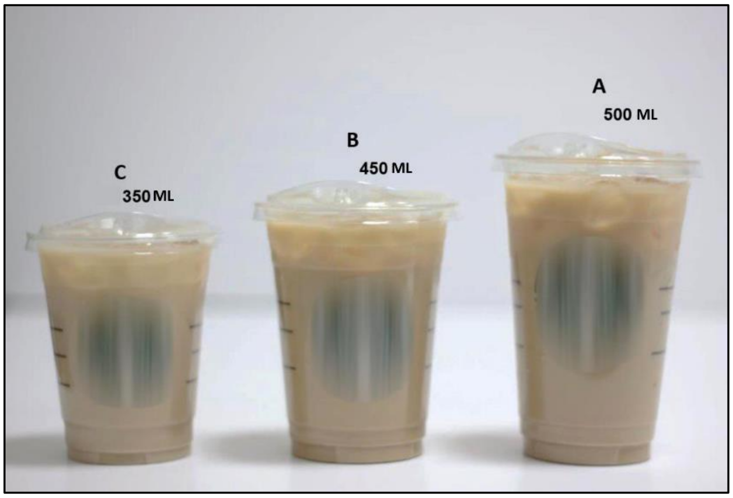

43/How often do you consume caramel macchiato, salted caramel, Frappuccino caramel or iced spinach latte?

- 6 or more times a day
- 5-4 times a day
- 3-2 times a day
- Once a day
- 5-6 times a week
- 4 -2 times a week
- Once a week
- Once to three times a month
- I don't consume

frontiers

44/What size do you usually consume from caramel macchiato, salted caramel, Frappuccino caramel or iced spinach latte?

- A
- B
- C

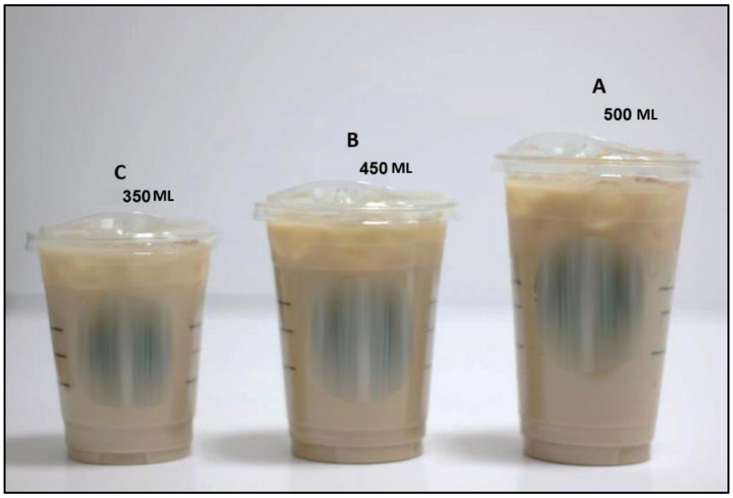

45/How often do you consume mocha or white chocolate mocha or Frappuccino mocha or iced white chocolate Frappuccino mocha?

- 6 or more times a day
- 5-4 times a day
- 3-2 times a day
- Once a day
- 5-6 times a week
- 4 -2 times a week
- Once a week
- Once to three times a month
- I don't consume

46/What size do you usually consume from mocha or white chocolate mocha or Frappuccino mocha or iced white chocolate Frappuccino mocha?

- A
- B
- C

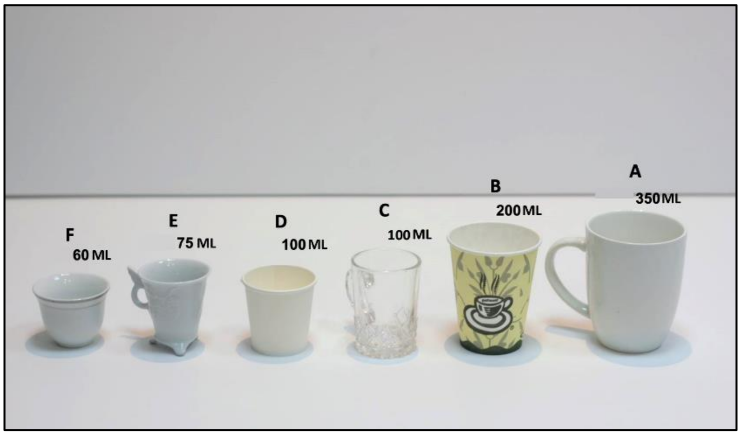

47/How often do you consume hot decaffeinated tea?

- 6 or more times a day
- 5-4 times a day
- 3-2 times a day
- Once a day
- 5-6 times a week
- 4 -2 times a week
- Once a week
- Once to three times a month
- I don't consume

48/What size do you usually consume from hot decaffeinated tea?

- A
- B
- C
- D
- E
- F

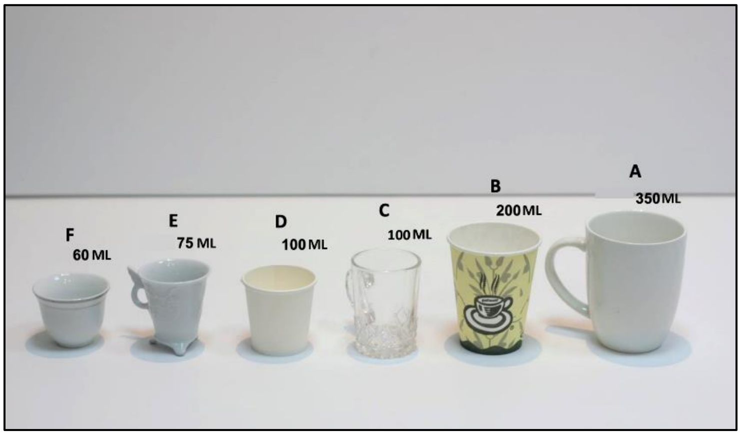

49/ How often do you consume hot red tea?

- 6 or more times a day
- 5-4 times a day
- 3-2 times a day
- Once a day
- 5-6 times a week
- 4 -2 times a week
- Once a week
- Once to three times a month
- I don't consume

50/What size do you usually consume from hot red tea?

- A
- B
- C
- D
- E
- F

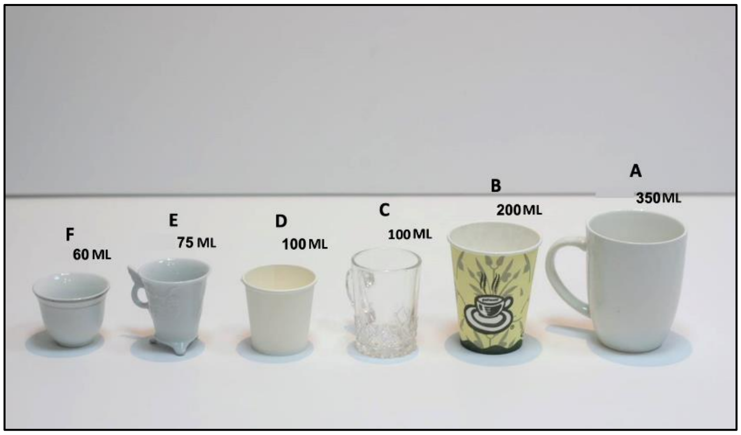

51/How often do you consume hot green tea?

- 6 or more times a day
- 5-4 times a day
- 3-2 times a day
- Once a day
- 5-6 times a week
- 4 -2 times a week
- Once a week
- Once to three times a month
- I don't consume

52/What size do you usually consume from hot green tea?

- A
- B
- C
- D
- E
- F

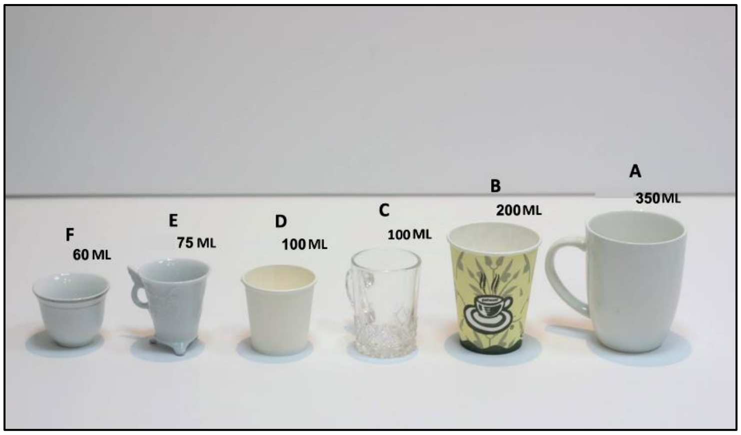

53/How often do you consume hot white tea?

- 6 or more times a day
- 5-4 times a day
- 3-2 times a day
- Once a day
- 5-6 times a week
- 4 -2 times a week
- Once a week
- Once to three times a month
- I don't consume

54/What size do you usually consume from hot white tea?

- A
- B
- C
- D
- E
- F

frontiers

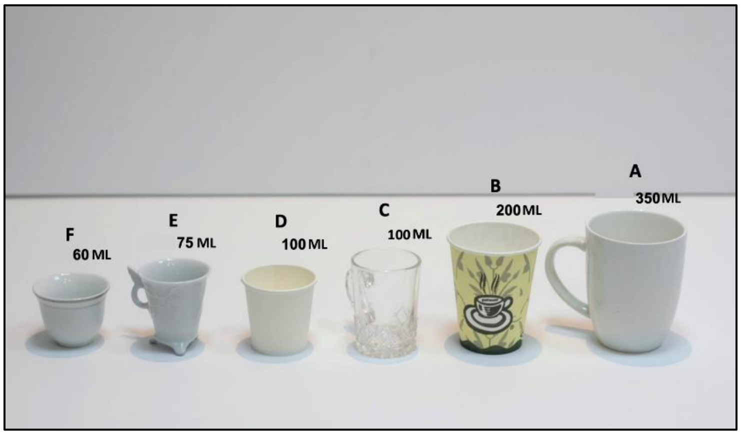

55/How often do you consume herbs (such as peppermint, basil, ginger, ... Etc.) hot?

- 6 or more times a day
- 5-4 times a day
- 3-2 times a day
- Once a day
- 5-6 times a week
- 4 -2 times a week
- Once a week
- Once to three times a month
- I don't consume

56/What size do you usually consume from herbs (such as peppermint, basil, ginger, ... etc.) hot?

- A
- B
- C
- D
- E
- F

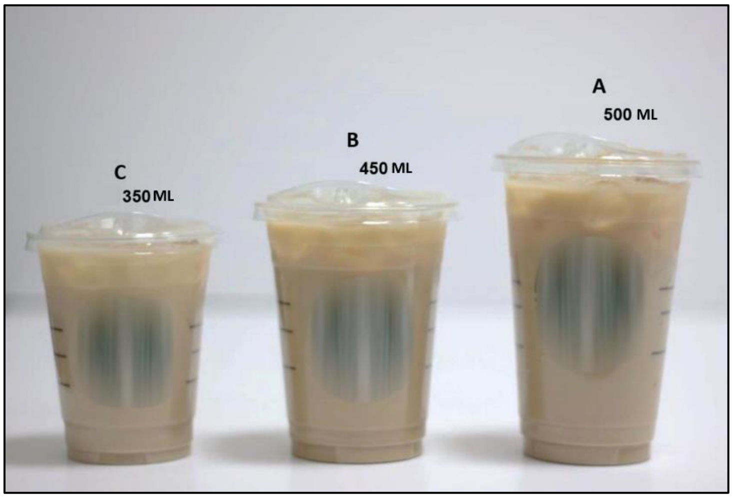

57/How often do you consume iced tea or iced tea with fruits?

- 6 or more times a day
- 5-4 times a day
- 3-2 times a day
- Once a day
- 5-6 times a week
- 4 -2 times a week
- Once a week
- Once to three times a month
- I don't consume

58/What size do you usually consume from iced tea or iced tea with fruits?

- A
- B
- C

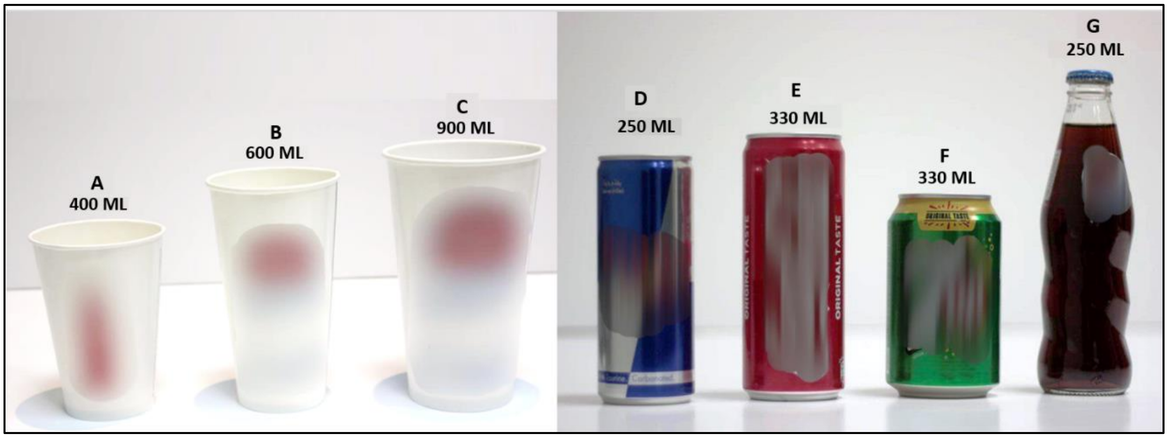

59/How often do you consume Code Red, bison, Red Bull or Power Horse?

- 6 or more times a day
- 5-4 times a day
- 3-2 times a day
- Once a day
- 5-6 times a week
- 4 -2 times a week
- Once a week
- Once to three times a month
- I don't consume

60/What size do you usually consume from Code Red, bison, Red Bull or Power Horse?

- A
- B
- C
- D
- E
- F
- G

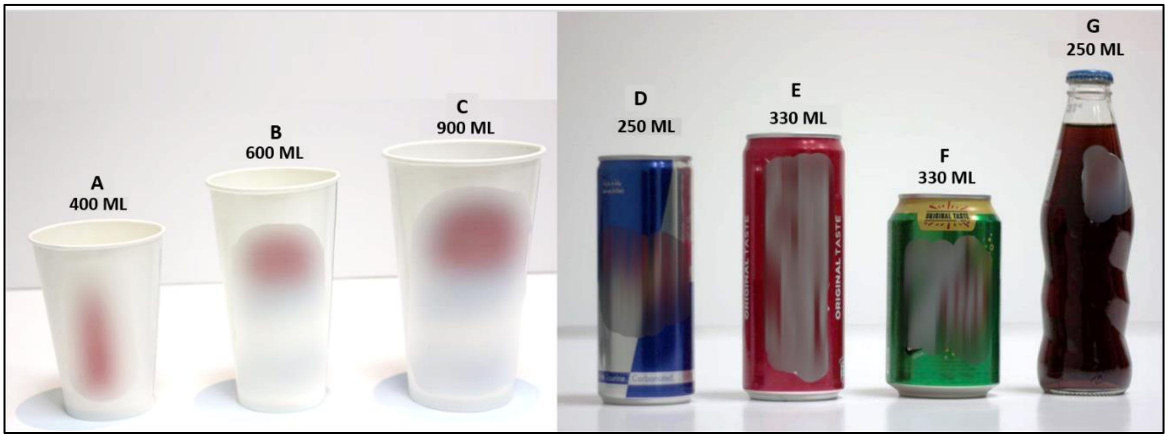

61/How often do you consume Pepsi Diet, Coca-Cola Zero or Mountain Dew Zero?

- 6 or more times a day
- 5-4 times a day
- 3-2 times a day
- Once a day
- 5-6 times a week
- 4 -2 times a week
- Once a week

- Once to three times a month
- I don't consume

62/What size do you usually consume from Pepsi Diet, Coca-Cola Zero or Mountain Dew Zero?

- A
- B
- C
- D
- E
- F
- G

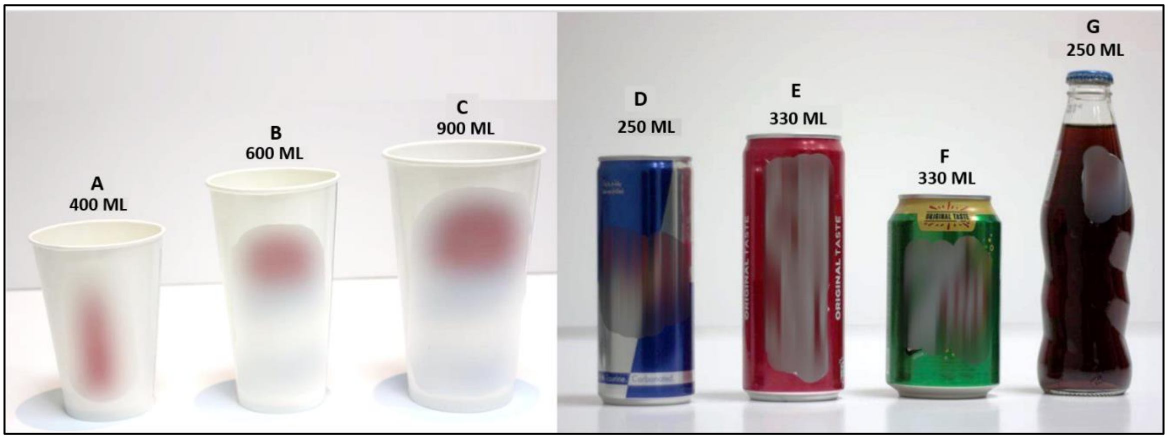

63/How often do you consume Pepsi, Coca-Cola or Mountain Dew?

- 6 or more times a day
- 5-4 times a day
- 3-2 times a day
- Once a day
- 5-6 times a week
- 4 -2 times a week
- Once a week
- Once to three times a month
- I don't consume

64/What size do you usually consume from Pepsi, Coca-Cola or Mountain Dew?

- A
- B
- C

- D
- E
- F
- G

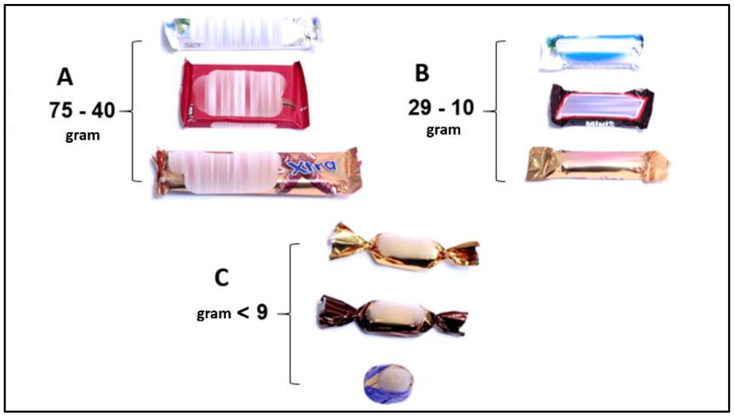

65/ How often do you consume dark chocolate?

- 6 or more times a day
- 5-4 times a day
- 3-2 times a day
- Once a day
- 5-6 times a week
- 4 -2 times a week
- Once a week
- Once to three times a month
- I don't consume

66/What size do you usually consume from dark chocolate?

- A
- B
- C

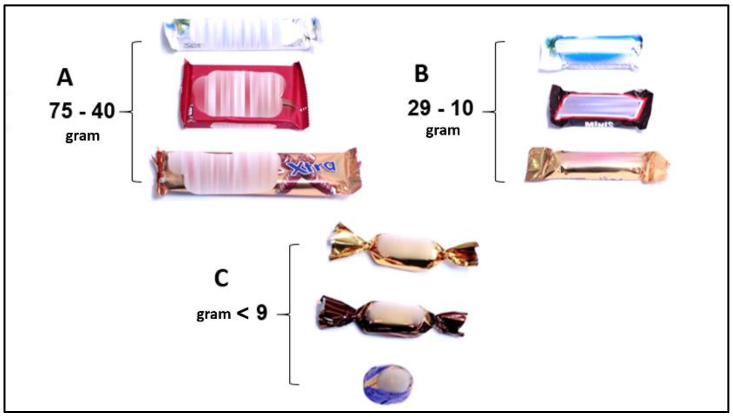

67/How often do you consume milk chocolate (such as: Twix, Snickers, Mars, Bounty or Kit Kat... Etc.)?

- 6 or more times a day
- 5-4 times a day
- 3-2 times a day
- Once a day
- 5-6 times a week
- 4 -2 times a week
- Once a week
- Once to three times a month
- I don't consume

68/What size do you usually consume from milk chocolate (such as Twix, Snickers, Mars, Bounty or Kit Kat... etc.)?

- A
- B
- C

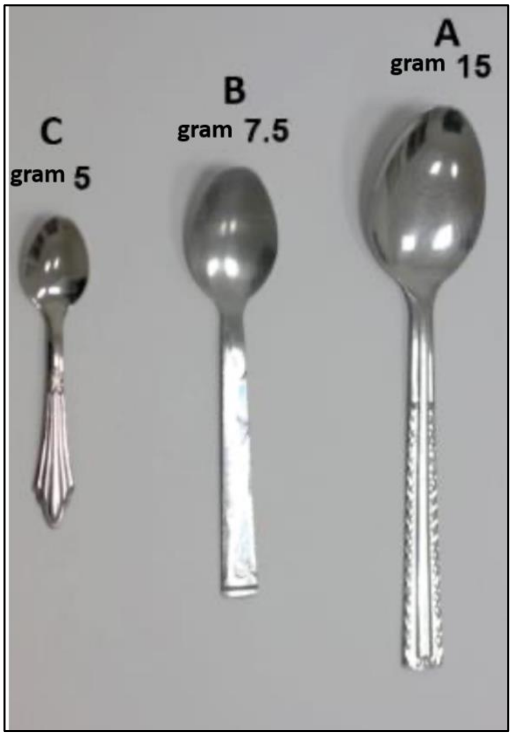

69/How often do you consume spreadable chocolate (e.g. Nutella or any other type)?

- 6 or more times a day
- 5-4 times a day
- 3-2 times a day

- Once a day
- 5-6 times a week
- 4 -2 times a week
- Once a week
- Once to three times a month
- I don't consume

70/What size do you usually consume from spreadable chocolate (e.g. Nutella or any other type)?

- A
- B
- C

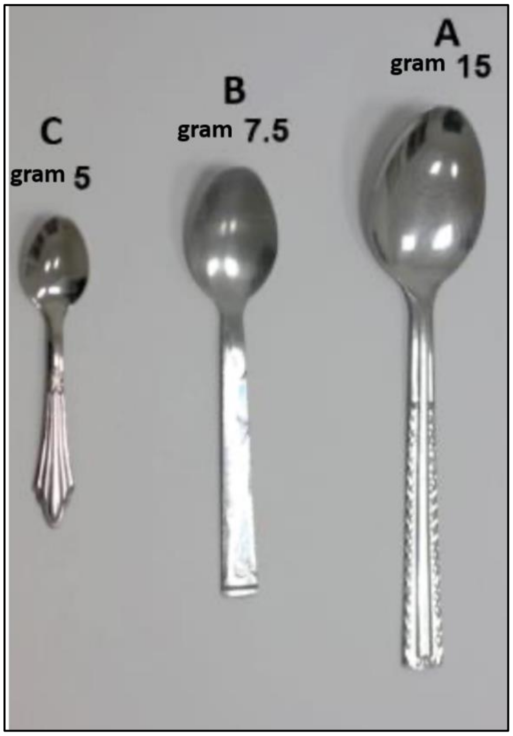

71/How often do you consume powdered chocolate?

- 6 or more times a day
- 5-4 times a day
- 3-2 times a day
- Once a day
- 5-6 times a week
- 4 -2 times a week
- Once a week
- Once to three times a month
- I don't consume

72/What size do you usually consume from powdered chocolate?

- A
- B
- C

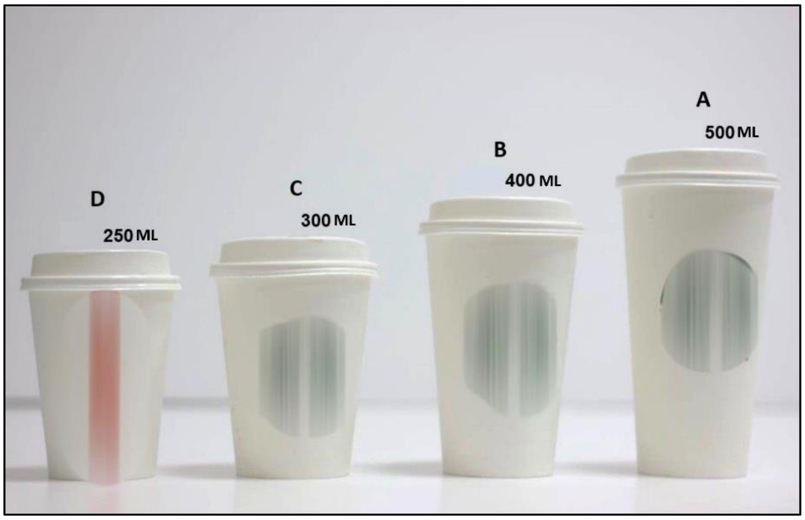

73/How often do you consume hot chocolate?

- 6 or more times a day
- 5-4 times a day
- 3-2 times a day
- Once a day
- 5-6 times a week
- 4 -2 times a week
- Once a week
- Once to three times a month
- I don't consume

74/What size do you usually consume from hot chocolate?

- A
- B
- C
- D

75/What are the reasons for your consumption of caffeine?
***(You can choose more than one answer)**

- To stay alert
- To increase focus
- To improve mood

- To avoid feeling tired
- To reduce the feeling of hunger
- For social courtesy
- Other ()

76/what time do you usually consume caffeine the most?
***(You can choose more than one answer)**

- Morning (6 am - 12 pm)
- Noon and afternoon (12 pm-6 pm)
- Evening (6pm-2am)
- Night (2 am - 6 am)

77/When you consume coffee drinks, do you add sugar or sweetener?

- Yes
- No
- I don't consume coffee

78/What kind of sweetener do you add to coffee drinks?
***(You can choose more than one answer)**

- White Sugar
- Brown Sugar
- Fruits Sugar (Fructose)
- Syrup (Maple syrup, caramel)
- Artificially Sweetener
- Honey

79/ Do you add milk to your coffee?

- Yes
- No

80/What kind of milk do you add to coffee drinks?
***(You can choose more than one answer)**

- Free Fat Milk
- Low-Fat Milk
- Whole-Fat Milk
- Evaporated Milk
- Condensed Milk
- Milk Substitutes

81/When you consume tea drinks, do you add sugar or sweetener?

- Yes
- No
- I don't consume tea

82/What kind of sweetener do you add to tea drinks?
***(You can choose more than one answer)**

- White Sugar
- Brown Sugar
- Fruits Sugar (Fructose)
- Syrup (Maple syrup, caramel)
- Artificially Sweetener
- Honey

83/When consuming tea drinks, do you add milk?

- Yes
- No
- I don't consume tea

84/What kind of milk do you add to tea drinks?
***(You can choose more than one answer)**

- Free Fat Milk
- Low-Fat Milk
- Whole-Fat Milk
- Evaporated Milk
- Condensed Milk
- Milk Substitutes

85/Do you think it is important that the content of food and beverages is displayed for caffeine on the food labels?

- Yes
- No

86/Do you think it is important that the content of food and beverages for caffeine is displayed on food and beverage menus for restaurants?

- Yes
- No

87/Do you think it is important to add the maximum limit of caffeine consumption on food and beverage menus for restaurants?

- Yes
- No
