# Supplementary material for: Caffeine consumption and exposure in Saudi Arabia: a cross-sectional analysis
Source: Front Nutr. 2025 May 20;12:1556001. doi: 10.3389/fnut.2025.1556001 (PMC12129793; doi:10.3389/fnut.2025.1556001)
Supplement: Supplementary file 2 [file Data_Sheet_2.pdf]

An overview of additional results

Table 1. Overview of food/beverage type, portion sizes, frequency option of Food Frequency Questionnaire on Caffeine Products (FFQ-C).

Category	Food/beverage type	Portion sizes	Frequency option
Coffee (15)	Saudi coffee, black coffee, Americano, Espresso, Cappuccino, Instant black coffee, Turkish coffee, Latte, iced black coffee, Caramel Macchiato or Spanish Latte, Mocha or White Mocha, Espresso con panna, Iced Latte, Iced Caramel Macchiato or salted Caramel or Caramel Frappuccino or Spanish Latte, Iced Mocha or White Mocha or Mocha Frappuccino or White Mocha Frappuccino.	60, 75, 100, 200, 250, 300, 350, 400, 450, and 500 ml.	Four categories per day (1 time, 2–3 times, 4–5 times, and > six times), three categories per week (1 time, 2–4 times, and 5–6 times), two categories per month (<1 time and 1–3 times), and not consumed.
Tea (6)	Decaffeinated Tea, Red Tea, Green Tea, Chai latte, Herbal tea (such as mint, ginger, etc.), and Iced tea with fruit.	60, 75, 100, 200, 350, 450, and 500 ml.	
Soft drinks (2)	Sweetened soft drinks and Diet soft drinks.	250, 330, 400, 600, and 900 ml.	
Energy drinks (1)	Energy drinks	250 and 330 ml.	

Chocolate (5)	Dark chocolate, chocolate with milk (Twix or Snickers or Mars or Bounty or Kit Kat), Spreadable chocolate (Nutella), chocolate powder, and Hot chocolate.	Hard<br><9 g, 10–29 g, and 40–75 g.<br>Spread<br>5, 7.5, and 15 g.<br>Drink<br>250, 300, 400, and 500 ml.	

Table 2. Caffeine content & reference information for caffeinated food and drink products (Coffee, Tea, Energy Drinks, Soft Drinks, and Chocolate).

Caffeine source	Sub-type	Caffeine content	Reference
Coffee	**Hot coffee**		
	Saudi Coffee	49.8 mg/100ml	SFDA-Lab[1] (Study)[2]
	Turkish Coffee	129.6 mg/100ml	SFDA-Lab[1] (Study)[2]
	Black Coffee	78.9 mg/100ml	companies & brands, websites[3]
	Black Coffee	77.2 mg/100ml	
	Americano Coffee	80.5 mg/100ml	
	Espresso	142.3 mg/100ml	(Study)[4]
	Coffee – Flavored with milk	44.9 mg/100ml	companies & brands, websites[3]
	Cappuccino	45.5 mg/100ml	
	Latte	40.4 mg/100ml	
	Caramel Macchiato or Spanish Latte	52.5 mg/100ml	
	Mocha or White Mocha	41.1 mg/100ml	
	**Iced Coffee**		
	Iced Black Coffee	78.1 mg/100ml	companies & brands, websites[3]
	Ice espresso or Espresso con panna	36 mg/100ml	companies & brands, websites[3]
	Iced Coffee – Flavored with milk	36.6 mg/100ml	companies & brands, websites[3]
	Iced Latte	33.3 mg/100ml	
	Iced Caramel Macchiato or salted Caramel or Caramel Frappuccino or Spanish Latte	38 mg/100ml	

	Iced Mocha or White Mocha or Mocha Frappuccino or White Mocha Frappuccino	38.6 mg/100ml	
**Tea**	Hot tea	31.6 mg/100ml	companies & brands, websites[3]
	Red Tea	45.1 mg/100ml	
	Green Tea	18.1 mg/100ml	
	Iced Tea	19.7 mg/100ml	
	Iced tea with fruit	4.5 mg/100ml	
**Soft Drinks**	Sugar-sweetened beverages	102.3 mg/100ml	companies & brands, websites[3]
	No sugar beverages	12.9 mg/100ml	
**Energy Drinks**	-	31.5 mg/100ml	companies & brands, websites[3]
**Chocolate**	**Solid Chocolate**		companies & brands, websites[3]
	Dark chocolate	56 mg/100mg	
	Chocolate with milk	20 mg/100mg	
	Spreadable chocolate	7 mg/100mg	
	Chocolate powder	230 mg/100mg	
	**Drink Chocolate**		
	Hot Chocolate	3.7 mg/100mg	

- [1]SFDA-Lab is the value calculated from the Saudi Food and Drug Authority Laboratory analysis.
- [2]The value from study (https://doi.org/10.3389/fnut.2024.1407590).
- [3] Companies & brands websites are the value calculated from the mean caffeine content of five different companies & brands websites.
- [4] The value from study (https://eprints.gla.ac.uk/58252/1/58252.pdf).

Table 3. Participants' frequency and percentage (%) of additive flavor choices among caffeine intake consumers (sweeteners and milk).

**Food Item**	**Coffee**		**Tea**	
**Additive**	**Frequency (*f*)**	**Percentage (%)**	**Frequency (*f*)**	**Percentage (%)**
**Sweeteners**				
White Sugar	182	70	522	98
Brown Sugar	24	9	10	2
Fruits Sugar (Fructose)	34	13	0	0
Syrup (Maple syrup, caramel)	5	2	0	0
Artificially Sweetener	16	6	0	0
Honey	0	0	0	0
**Total**	261	100	532	100
**Milk**				
Free Fat Milk	28	5	6	32
Low-Fat Milk	152	26	10	53
Whole-Fat Milk	290	50	1	5
Evaporated Milk	55	9	1	5
Condensed Milk	21	4	1	5
Milk Substitutes	39	7	0	0
**Total**	585	100	19	100

Table 4: Community perceptions on caffeine related food/drinks legislation.

Question	Answer	Number of participants (N)	Percentage (%)
Do you think it is important that the content of food and beverages is displayed for caffeine on the food labels?	Yes	951	92
Do you think it is important that the content of food and beverages for caffeine is displayed on food and beverage menus for restaurants?	Yes	949	91
Do you think it is important to add the maximum limit of caffeine consumption on food and beverage menus for restaurants?	Yes	967	9

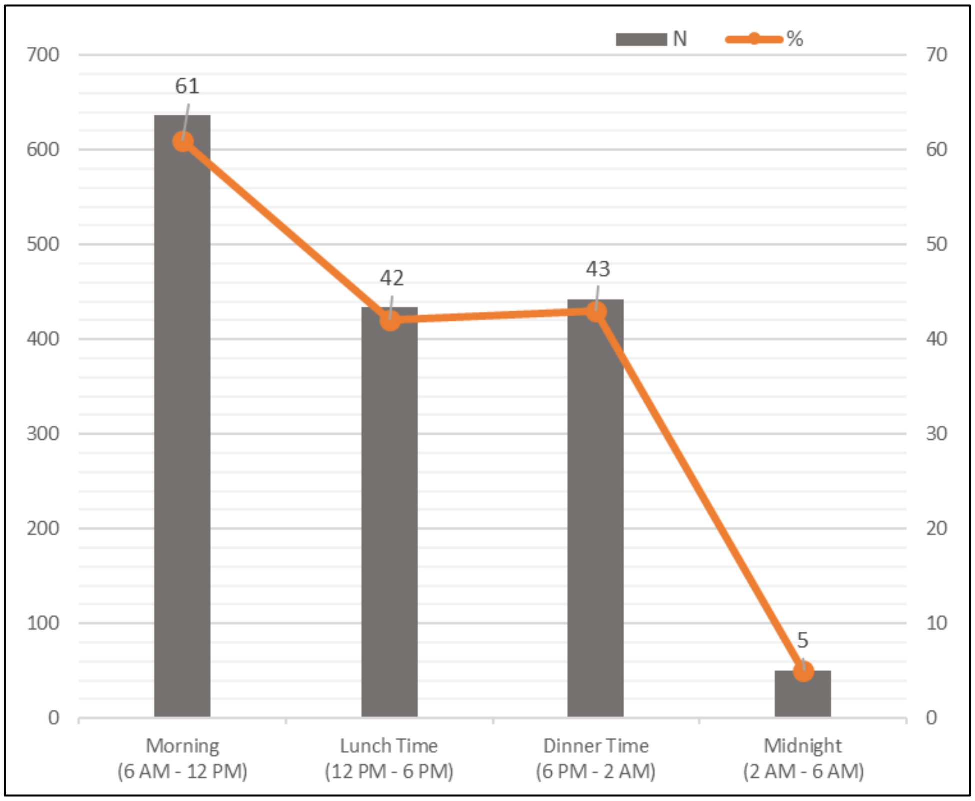

Figure 1: Participants' daily period of caffeine consumption during morning, lunch time, dinner time, and midnight.
